# Artificial Intelligence in Clinical Oncology: From Productivity Enhancement to Creative Discovery

**DOI:** 10.3390/curroncol32110588

**Published:** 2025-10-22

**Authors:** Masahiro Kuno, Hiroki Osumi, Shohei Udagawa, Kaoru Yoshikawa, Akira Ooki, Eiji Shinozaki, Tetsuo Ishikawa, Junna Oba, Kensei Yamaguchi, Kazuhiro Sakurada

**Affiliations:** 1 Department of Gastroenterological Chemotherapy, Cancer Institute Hospital, Japanese Foundation for Cancer Research, Tokyo 135-8550, Japan; masahiro.kuno@jfcr.or.jp (M.K.); shohei.udagawa@jfcr.or.jp (S.U.); kaoru.yoshikawa@jfcr.or.jp (K.Y.); akira.oki@jfcr.or.jp (A.O.); eiji.shinozaki@jfcr.or.jp (E.S.); kensei.yamaguchi@jfcr.or.jp (K.Y.); 2 The Ishii-Ishibashi Laboratory, Department of Extended Intelligence for Medicine, Graduate School of Medicine, Keio University School of Medicine, Tokyo 160-8582, Japan; tetsuo.ishikawa@keio.jp (T.I.); joba@keio.jp (J.O.); 3 Predictive Medicine Special Project (PMSP), RIKEN Center for Integrative Medical Sciences (IMS), RIKEN, Yokohama 230-0045, Japan; 4 Division of Applied Mathematical Science, RIKEN Center for Interdisciplinary Theoretical and Mathematical Sciences (iTHEMS), RIKEN, Yokohama 230-0045, Japan; 5 Collective Intelligence Research Laboratory, Graduate School of Arts and Sciences, The University of Tokyo, Tokyo 153-8902, Japan

**Keywords:** artificial intelligence, machine learning, deep learning, foundation models, large language model, retrieval-augmented generation, clinical oncology, precision medicine, FUTURE-AI framework

## Abstract

**Simple Summary:**

Artificial intelligence (AI) is transforming cancer care by analyzing vast amounts of medical data that exceed human capacity. This review examines how AI enhances both productivity and creativity in clinical oncology. AI improves productivity by automating routine tasks like image analysis and report generation, allowing doctors to focus on patient care. More importantly, AI enables creative discovery by finding hidden patterns across different data types—combining pathology images, radiology scans, and genetic information to identify new biomarkers and treatment approaches. The FUTURE-AI framework provides practical guidelines to ensure these AI tools are fair, reliable, and understandable when used in clinical practice. This paper highlights how AI augments rather than replaces physician expertise, ultimately improving cancer diagnosis and treatment.

**Abstract:**

Modern clinical oncology faces an unprecedented data complexity that exceeds human analytical capacity, making artificial intelligence (AI) integration essential rather than optional. This review examines the dual impact of AI on productivity enhancement and creative discovery in cancer care. We trace the evolution from traditional machine learning to deep learning and transformer-based foundation models, analyzing their clinical applications. AI enhances productivity by automating diagnostic tasks, streamlining documentation, and accelerating research workflows across imaging modalities and clinical data processing. More importantly, AI enables creative discovery by integrating multimodal data to identify computational biomarkers, performing unsupervised phenotyping to reveal hidden patient subgroups, and accelerating drug development. Finally, we introduce the FUTURE-AI framework, outlining the essential requirements for translating AI models into clinical practice. This ensures the responsible deployment of AI, which augments rather than replaces clinical judgment, while maintaining patient-centered care.

## 1. Introduction

Modern clinical oncology is at a turning point, shaped by accumulating biological knowledge and growing machine computing power. Cancer care is becoming more personalized through rapid advances in precision diagnostics and targeted therapies. However, this progress has also produced a vast, heterogeneous body of patient data that exceeds clinicians’ capacity to analyze and interpret [1,2]. High-resolution imaging, digital pathology, and multilayered genomic, transcriptomic, and proteomic profiles now require new analytic approaches [3,4,5]. In this context, artificial intelligence (AI) is not a future prospect but rather a present necessity for improving cancer care.

The integration of AI into clinical oncology is propelled by two related forces. First, breakthroughs in computational methods, particularly deep learning (DL), have produced multilayer artificial neural networks capable of detecting subtle patterns in extensive biomedical datasets that often escape human perception. Second, the digital transformation of healthcare has matured, with routine electronic health records (EHRs), whole-slide imaging (WSI) in pathology, and standardized genomic profiling now yielding longitudinal, high-quality datasets for training and validating AI models [6,7,8,9,10,11]. This creates a self-reinforcing data–AI loop: expanding datasets enable better models, whose finer-grained outputs become new data, further increasing the need for intelligent systems.

The impact of AI in oncology can be framed along two axes: productivity and creativity. AI improves productivity by automating labor-intensive tasks, reducing diagnostic errors and streamlining clinical workflows. AI also enables creativity by uncovering new biological insights, generating hypotheses, and supporting diagnostic and therapeutic strategies that were previously infeasible. This review examines how AI reshapes clinical workflows, enables novel research, and extends precision medicine through this lens. It outlines core technologies alongside the technical, ethical, and operational barriers to implementation.

## 2. An Overview of AI Technologies and Their Relevance to Oncology

**Figure 1:**
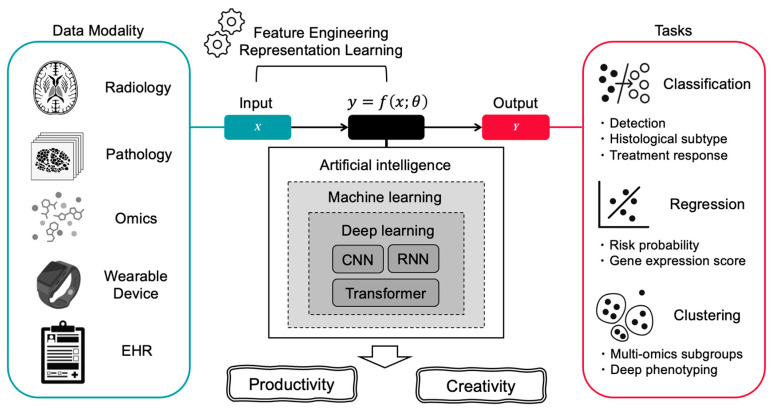
Overview of AI Technologies and Their Relevance to Oncology. This figure illustrates how artificial intelligence transforms diverse clinical data sources into actionable insights for cancer care. The left panel shows various data modalities including radiology images, digital pathology slides, multi-omics data, wearable device outputs, and electronic health records (EHR). These inputs are processed through AI’s core components: representation learning powered by traditional ML and DL. The AI system achieves two primary outcomes: productivity enhancement (including automated detection, classification, differential subtype analysis, treatment response prediction, and regression tasks such as gene expression scoring) and creative discovery (enabling unsupervised clustering, multi-omics subgroup identification, and deep phenotyping).

**Figure 2:**
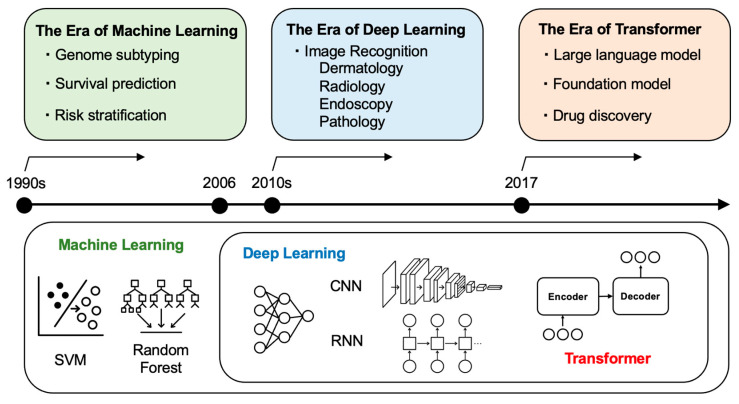
AI Evolution and Application in Oncology: From Machine Learning to Transformers. Timeline showing the technological progression of AI in oncology across three eras: the era of machine learning (1990s) characterized by genome subtyping, survival prediction, and risk stratification using traditional algorithms (SVM, Random Forest); the era of deep learning (2010s–) featuring image recognition in dermatology, radiology, endoscopy, and pathology using CNN and RNN architectures; and the era of transformers (2017–present) enabling large language models, foundation models, and drug discovery applications.

AI in oncology has evolved through several distinct technological eras, each introducing new capabilities for data interpretation, prediction, and clinical decision support (Figure 1 and Figure 2). AI has progressed from traditional machine learning (ML) in the 1990s to deep learning (DL) in the 2010s and, since 2017, to transformer-based foundation models. During this period, the focus of analysis has shifted from hand-crafted features on structured data (data organized in tables with rows and columns, like blood test results or patient demographics) to representation learning for unstructured and multimodal inputs. This approach automatically learns features and latent vectors (embeddings) from data, reducing the need for manual feature engineering and providing reusable representations for downstream tasks such as classification, regression, and clustering, introducing generative methods (Figure 1).

ML forms the foundation of modern AI. Since the 1990s, ML has allowed computers to learn from examples rather than from handwritten rules for each task. In oncology, traditional ML models, such as support vector machines (SVM), decision trees, and random forests, have supported tumor classification, survival prediction, and risk stratification using structured clinical or genomic data [6,12,13,14]. However, a key limitation is the need for manual feature engineering, which requires domain expertise and limits scalability for unstructured inputs.

DL emerged in the 2010s, replacing hand-crafted features with networks that learn hierarchical representations from raw inputs. These multilayer neural networks capture complex, non-linear patterns in high-dimensional data. In oncology, DL has advanced image analysis in fields such as dermatology, radiology, endoscopy, and pathology. Convolutional neural networks (CNNs), built for spatial data, have demonstrated performance comparable to that of specialists in cancer detection and tumor segmentation [15,16,17,18,19,20]. For sequential data, recurrent neural networks (RNNs) support modeling of clinical time series and genomic sequences [20,21,22].

Among DL architectures, the transformer (introduced in 2017 and used in tools such as ChatGPT) first achieved breakthrough results in machine translation between languages and has since been generalized to a wide range of sequence modeling tasks, becoming a major driver of recent advances in AI [23]. A transformer uses self-attention: for each element in the input, the model weighs how much all other elements should influence it, creating context-aware representations and capturing long-range relationships. Since text, images, and genomic sequences can all be broken into ordered pieces, the same method can be applied beyond language. In oncology, transformer-based methods support clinical text processing, whole-slide and radiologic image interpretation (vision transformers), and genomic sequence modeling [24,25,26,27]. As models and datasets grew larger, this same architecture gave rise to “foundation models”—large transformers pre-trained on broad data and adaptable to many tasks [28].

In summary, AI in oncology has evolved from hand-crafted, task-specific algorithms to large, adaptive platforms that handle multimodal data and produce clinically relevant outputs (Figure 1). Traditional ML remains useful for small, well-curated datasets, whereas DL and transformer-based models address increasingly complex problems. These technologies are now being applied across clinical oncology. The following sections provide concrete examples of how these technologies improve productivity and support creativity.

## 3. Productivity Enhancement in Clinical Oncology

In clinical oncology, the main benefit of AI is increased productivity. AI can automate routine tasks, promote diagnostic consistency, and analyze large datasets. When used as assistive tools, these systems streamline workflows and allow clinicians to focus on complex decisions and patient care. This section reviews applications in three domains: imaging-based diagnostic support; natural language processing and large language models (LLMs) for documentation and data structuring; and AI tools that accelerate research workflows.

### 3.1. AI for Image-Based Oncologic Diagnosis: Radiology, Digital Pathology, Endoscopy, and Dermatology

As of 2024, the FDA had cleared or approved approximately 700 AI/ML-enabled medical devices, 76% of which are related to radiology. Most of these devices target image analysis, with oncologic imaging as a major application [1,29]. In mammography, deep learning systems have achieved radiologist-comparable breast cancer detection. In Sweden’s randomized Mammography Screening with Artificial Intelligence (MASAI) trial, 105,934 women were assigned to either AI-supported screening or standard double reading; the AI-supported screening was non-inferior and reduced radiologist workload by 44.2% (AI arm, 61,248 reads; control, 109,692) without increasing false-positive rates, and with evidence of earlier cancer detection [30,31].

Pathology, which is essential for definitive cancer diagnosis, has adopted imaging through digital pathology [32,33,34,35,36]. A recent meta-analysis that pooled 100 studies using more than 152,000 WSI reported a mean sensitivity of 96.3% and a mean specificity of 93.3%. Performance varied by subspecialty: gastrointestinal (93%/94%), urologic (95%/96%), and breast (83%/88%) [35]. Foundation-model efforts are emerging as evidenced by the GigaPath project, a collaboration among Microsoft, Providence Health System, and the University of Washington, which pretrained Prov-GigaPath on more than 170,000 slides (>1 billion 256 × 256-pixel tiles) and is among the first open-access foundation models for digital pathology; it has shown strong performance in cancer classification, mutation prediction, and vision-language tasks [36].

In addition to radiology and pathology, image-based AI has proven useful in endoscopy and dermatology. Unlike other modalities, endoscopy requires real-time analysis of video streams [37,38,39,40,41,42]. In colonoscopy, AI-assisted systems have increased the adenoma detection rate (ADR) in randomized trials through real-time polyp identification. Meta-analyses report a ~14% relative improvement in the ADR over standard colonoscopy [39,40]. In dermatology, a foundation model such as PanDerm, trained on >2 million dermoscopic and clinical images, improved physicians’ diagnostic accuracy for skin cancer by >10%, with the greatest benefits observed for non-dermatologists [26].

### 3.2. NLP for Data Structuring and Report Generation

Natural language processing (NLP) increases productivity in clinical oncology by converting unstructured text into analyzable data and standardizing documentation [41,42,43,44,45,46]. Oncology generates large volumes of free-text notes, including treatment histories, adverse events, response assessments, and progression narratives, that are difficult to query at scale. NLP methods help structure these records for quality improvement and research purposes.

Recent medically specialized LLMs address these needs. Models such as Med-PaLM, Clinical-T5, and GatorTron are trained on medical corpora and show stronger understanding of clinical text than general-purpose models [46,47,48,49]. Notably, Woollie has been reported as an oncology-specific LLM with multi-institutional validation: trained on real-world data from Memorial Sloan Kettering Cancer Center across lung, breast, prostate, pancreatic, and colorectal cancers, it achieved an area under the receiver operating characteristic curve (AUROC) of 0.97 for progression prediction, with external validation at University of California, San Francisco (UCSF) showing an AUROC of 0.88 [50]. While this performance drop indicates challenges in generalization across institutions, the model maintains clinically relevant predictive capability, demonstrating the importance of diverse training data and rigorous external validation.

Generative AI is also being used for radiology report drafting [51,52,53,54,55]. These systems can generate structured reports based on imaging findings, standardize terminology, and guarantee the inclusion of critical elements, such as tumor measurements and response assessments, according to Response Evaluation Criteria in Solid Tumors (RECIST) guidelines. This automation reduces reporting variability and improves consistency in evaluating treatment responses across different radiologists and institutions. One study reported a 30% reduction in initial report generation time, though comprehensive evaluation including verification and correction time by radiologists remains limited [56].

Finally, AI supports clinical trial operations by automating patient-protocol matching. Systems parse biomarker requirements, prior therapies, and performance status from EHRs, identify potentially eligible patients, and flag candidates for screening [56,57,58,59,60,61,62]. Programs using real-time matching from automatically extracted criteria have reported up to a 50% increase in enrollment in precision oncology trials [57]. Integration with e-consent and automated pre-screening is expected to further streamline recruitment and expand access.

### 3.3. AI-Powered Research Support Tools

**Figure 3:**
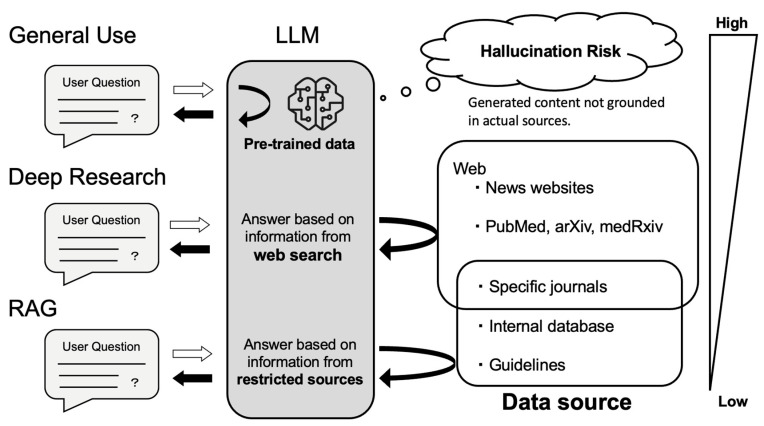
How LLMs Answer Questions: Source Dependency and Hallucination Risk. General use relies solely on pre-trained data, which may result in hallucinations when content is not grounded in actual sources. Deep research mode performs real-time searches across openly available sources such as news websites, PubMed, arXiv, and medRxiv, allowing access to more recent but not necessarily curated information. Retrieval-augmented generation (RAG) answers questions based on specified, high-quality sources—including specific journals, internal databases, and clinical guidelines—thus providing the most reliable responses by constraining outputs to verified and authoritative content.

AI improves research productivity by making literature searches more efficient and by accelerating data analysis, allowing investigators to spend more time on hypothesis generation and interpretation rather than manual screening and coding. General-purpose, LLM-based chat systems (e.g., ChatGPT, Gemini) can answer academic questions from their pretraining data (Figure 3). Hallucination remains a critical safety concern in healthcare applications. LLM-based chat systems can generate plausible-sounding but factually incorrect information with high confidence, potentially leading to dangerous clinical decisions. Newer “deep research” or web-enabled modes address this issue by running targeted searches at query time, gathering recent sources, and returning cited summaries. In practice, these modes offer a faster and more comprehensive starting point for literature reviews than manual keyword searches alone. However, coverage and citation quality still vary by platform and query, so source verification is still essential.

Reliability can be strengthened further with retrieval-augmented generation (RAG), which grounds model outputs in documents fetched from trusted corpora (e.g., PubMed, guidelines, institutional repositories) [63,64]. Conceptually, RAG follows three steps: retrieving passages relevant to the question; augmenting the prompt with those excerpts and bibliographic metadata; and generating an answer that quotes or cites the retrieved sources. Because the model is constrained to what it has “in context,” factual drift is less likely. Performance depends on corpus quality and retrieval ranking. Medical tools such as OpenEvidence use this approach [65]. The American Society of Clinical Oncology (ASCO) Guideline Assistant similarly restricts responses to curated ASCO content, improving reliability by design [66]. While RAG reduces the risk of hallucination, it does not eliminate the risk if retrieved content is incomplete or incorrect. This is why human verification of all outputs remains essential, particularly for clinical decision-making.

Beyond search, LLMs lower the barrier to quantitative work by translating plain-language requests into statistical code or running analyses directly [67]. Tasks that once required SPSS/SAS expertise or R/Python coding (e.g., survival analysis, multivariable regression, and subgroup analyses) can now begin with prompts such as “compare progression-free survival between arms, adjusting for age and performance status.” This “vibe coding” workflow—iteratively stating the intent of the analysis and having the system draft or revise code—helps clinician-researchers prototype quickly [68]. While vibe coding offers remarkable convenience, it carries substantial risks when users lack fundamental statistical and analytical knowledge. Outputs still require method review (e.g., assumptions, model choice, and multiple testing), reproducibility safeguards (version-controlled code, fixed random seeds, environment capture), and appropriate governance for patient data.

Although commercial AI tools are useful, clinicians must exercise caution when entering medical information into chat interfaces. Avoid submitting protected health information or potentially identifying details to public or consumer services. When clinical use is required, rely on institution-approved enterprise deployments with formal data-processing agreements, role-based access control, audit logging, and explicit data-retention settings (including disabling provider training on inputs) [69]. Prefer de-identified or synthetic data in prompts, and, when feasible, keep retrieval and model inference on-premises or within a virtual private cloud under institutional governance.

## 4. Creative Discovery in Clinical Oncology

While productivity gains are important, AI’s greater impact in oncology may be its ability to enable new discoveries. By analyzing complex, high-dimensional data, AI moves beyond optimizing existing tasks and supports the identification of previously unrecognized biological relationships and the design of new diagnostic and treatment approaches. This section examines how AI contributes to creative discovery in precision oncology.

### 4.1. Computational Biomarkers for Precision Oncology

AI is reshaping biomarker development by shifting the focus from single-analyte markers to computational biomarkers—patterns learned from combinations of images, molecular profiles, and clinical data. Traditional efforts focused on a specific protein or mutation linked to disease or treatment response, and AI extends this by modeling non-linear interactions across modalities to capture tumor biology more completely.

**Figure 4:**
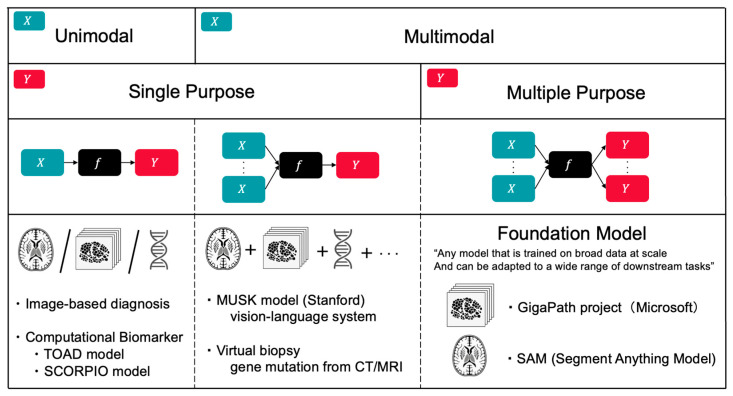
Classification of AI Models by Input Modality and Output Task Multiplicity. Framework for categorizing AI models in oncology based on data modality (unimodal vs. multimodal) and task scope (single vs. multiple purpose). Examples include image-based diagnosis and computational biomarkers (TOAD, SCORPIO) for unimodal applications; MUSK model (Stanford vision-language system) and virtual biopsy for multimodal single-purpose tasks; and foundation models (GigaPath, SAM) that leverage multimodal inputs for multiple downstream applications. This classification illustrates the evolution toward integrated, versatile AI systems in precision oncology.

The most active line of work is multimodal deep learning (MDL), which integrates histopathology (“pathomics”), radiology (“radiomics”), genomics, and clinical records to create unified patient-level representations [70,71,72] (Figure 4). The Multimodal transformer with Unified maSKed modeling (MUSK) from Stanford exemplifies this approach as a vision-language system that pretrains on large, largely unpaired corpora of pathology image patches and clinical text, then aligns image–text pairs for downstream tasks [32]. In evaluations of lung and gastroesophageal cancers, MUSK outperformed the standard programmed cell death ligand 1 (PD-L1) biomarker alone in predicting immunotherapy benefit and surpassed clinicopathologic baselines for survival-related endpoints. These gains reflect the value of combining visual pathological features with contextual information from EHRs.

A useful distinction can be made between noninvasive “virtual biopsies” and histology-derived molecular surrogates [5,33,73]. Virtual biopsy refers to inferring histologic or genomic attributes directly from clinical imaging without tissue sampling—for example, predicting epidermal growth factor receptor (EGFR) mutation status from baseline computed tomography (CT) in non-small cell lung cancer or isocitrate dehydrogenase (IDH) mutation/1p/19q codeletion from brain magnetic resonance imaging (MRI) [74,75,76]. By contrast, histology-derived surrogates use hematoxylin and eosin (H&E) slides to infer molecular features (e.g., microsatellite instability (MSI) in colorectal cancer or Oncotype DX–like recurrence scores in breast cancer) [77,78,79,80,81]. The former may reduce the need for an initial biopsy; the latter can reduce add-on assays and cost while still relying on tissue obtained for standard care. Both require prospective, multi-site validation before routine adoption.

Beyond histology-derived surrogates, computational biomarkers with the potential to influence treatment decisions are emerging. The TOAD (Tumour Origin Assessment via Deep learning) model estimates tissue of origin from routine H&E WSIs [82]. Trained on 17,486 slides spanning 18 primary sites, it achieved top-1/top-3 accuracies of approximately 0.83/0.96 on an internal test set and 0.80/0.93 on an external test set; in a curated cancer of unknown primary (CUP) cohort (*n* = 317), its top-three predictions matched pathologists’ differentials in 82% of cases. By providing slide-level attention maps and calibrated probabilities, TOAD can prioritize ancillary testing and assist in the diagnostic workup. Another example is the SCORPIO (Standard Clinical and labOratory featuRes for Prognostication of Immunotherapy Outcomes) model, which predicts immunotherapy outcomes using only routine laboratory panels (complete blood counts and metabolic profiles) and basic clinical variables [83]. Developed on an institutional cohort (*n* ≈ 1600) and validated across internal sets (*n* ≈ 2500), multicenter phase-3 trials (*n* ≈ 4400), and an external real-world cohort (*n* ≈ 1100), the SCORPIO model covers nearly 10,000 immune checkpoint inhibitors (ICI)-treated patients across more than 20 types of cancers. SCORPIO outperformed tumor mutational burden (TMB) and PD-L1 in predicting overall survival and clinical benefit, offering a low-cost risk stratifier that can complement genomic tests where access is limited.

### 4.2. Unsupervised Discovery and Deep Clinical Phenotyping

Clinical courses in cancer vary widely across patients. This heterogeneity reflects diversity within tumor cells as well as germline genetic factors and environmental influences. To manage these differences, patient stratification has long been a core goal. Early approaches relied on gross morphology—histologic subtype—and staging systems such as TNM (and FIGO for gynecologic malignancies). These frameworks, derived from surgical and pathologic assessments, have guided decisions about extent of disease and initial management. Over time, molecular classification based on driver alterations has become central to treatment. Examples include selecting targeted therapies for EGFR-mutant lung cancer or human epidermal growth factor receptor 2 (HER2)-positive breast cancer [84,85,86].

Machine learning enables data-driven stratification that may reveal patterns not easily recognized by clinicians. In a 2019 study from RIKEN and Jikei University, investigators analyzed 435 patients with ovarian tumors using age and 32 preoperative laboratory measurements [87]. A supervised model distinguished malignant from benign tumors with an AUROC of 0.968. Applying unsupervised clustering to the same variables identified two groups within early-stage ovarian cancer: one with laboratory patterns resembling benign disease (cluster 1) and another resembling advanced cancer (cluster 2). Cluster 1 showed very low recurrence, whereas cluster 2 had higher recurrence and mortality, indicating a previously unrecognized subtype linked to prognosis based solely on preoperative data.

Recent work combines high-dimensional omics with DL to define subtypes grounded in tumor biology or hidden tissues structures. In hepatocellular carcinoma, for example, proteogenomic studies integrating whole-genome sequencing, RNA sequencing, and quantitative proteomics have identified three molecular classes: immune-“hot” with favorable outcomes, proliferative and TP53-enriched with angiogenic signaling and poorer outcomes, and CTNNB1-enriched with mTOR-pathway activation [88,89,90]. Candidate protein markers emerging from these analyses suggest targets for risk stratification and therapy development. Spatial omics extends phenotyping by mapping cell types and their interactions in situ [91,92,93,94]. Spatial transcriptomic analyses of lung adenocarcinoma show that, as tumors progress from in situ to invasive stages, the tumor microenvironment shifts from a relatively balanced immune milieu to a more immunosuppressive landscape. Incorporating such spatial information supports classification along immune context (often summarized as “hot” versus “cold” tumors).

Complementing these tumor-focused approaches, deep phenotyping emphasizes comprehensive, longitudinal profiling of individual physiology beyond the tumor itself [95,96,97,98]. This strategy integrates genomic sequencing, blood-based omics (e.g., metabolomics and proteomics), microbiome analysis, and wearable device data to capture each patient’s unique biological state over time. Importantly, while medicine has accumulated vast knowledge about individual causal mechanisms through experiments and clinical trials, integrating these fragmented insights remains challenging. AI for Medicine addresses this by combining data-driven models with domain knowledge—incorporating biological pathways and causal constraints into AI architectures rather than treating them as black boxes. This hybrid approach enables discovery of novel patterns while respecting established mechanistic understanding. By monitoring multiple physiological scales simultaneously, deep phenotyping can detect early transitions from wellness to disease and inform personalized treatment decisions based on the individual’s complete biological context rather than tumor characteristics alone. This holistic approach represents a paradigm shift toward predictive, preventive, personalized, and participatory (P4) medicine, which could transform cancer care by enabling earlier intervention and more precise therapeutic matching [99,100]. By quantifying disease heterogeneity and predicting individual trajectories, AI-enhanced deep phenotyping moves us closer to the dual goals of precision oncology: optimal treatment selection for existing cancers and proactive identification of at-risk individuals for prevention.

### 4.3. Drug Discovery

Drug discovery has long been slow, costly, and associated with high attrition rates. A recent breakthrough, DeepMind’s AlphaFold, addressed the decades-old “protein-folding problem” and won the 2024 Nobel Prize in Chemistry [101]. By accurately predicting three-dimensional structures from sequences at the proteome scale, AlphaFold removes a frequent bottleneck in structure-based design and makes structural hypotheses more accessible. Building on this progress, deep generative models now design novel small molecules while predictive models triage candidates, forming a computational pipeline from target structure to testable chemical matter.

An end-to-end example has been reported for hepatocellular carcinoma. Using an integrated platform (Pharma.AI), disease-gene analyses identified CDK20 as a target (PandaOmics), an AlphaFold2 model supplied the target structure, and a generative module (Chemistry42) produced approximately 9000 candidate molecules [102,103]. Seven of these molecules were synthesized and tested; one showed promising activity in liver cancer cells and advanced as a lead, all within roughly 30 days from design initiation. A second example concerns WSB1, a protein linked to metastasis through hypoxia-response pathways in lung and pancreatic cancers but historically underexplored as a drug target [104]. Investigators built an AlphaFold2 model of WSB1, refined it with molecular simulation, and conducted virtual screening across millions of compounds. Approximately 20 candidates were selected for synthesis and testing, yielding four lead molecules. These cases demonstrate how structure prediction and large-scale in silico triage can produce tractable chemical matter for previously neglected targets.

AlphaFold has been a breakthrough for drug discovery, including cancer therapeutics, but its limitations are well recognized [105,106,107,108]. It predicts a protein’s most stable shape; however, it does not model how the protein moves. Drug binding often depends on motion, such as small shifts when a compound binds, long-range effects within the protein, hidden pockets that open and close, or changes when proteins form complexes. Cancer-related mutations and chemical modifications can also alter these shapes. Therefore, drug development pipelines should incorporate computer simulations of protein motion, test binding against several plausible protein shapes, and confirm results with laboratory assays before moving candidates forward.

## 5. From Algorithm to Bedside: A FUTURE-AI–Informed Guide for Oncologists

**Figure 5:**
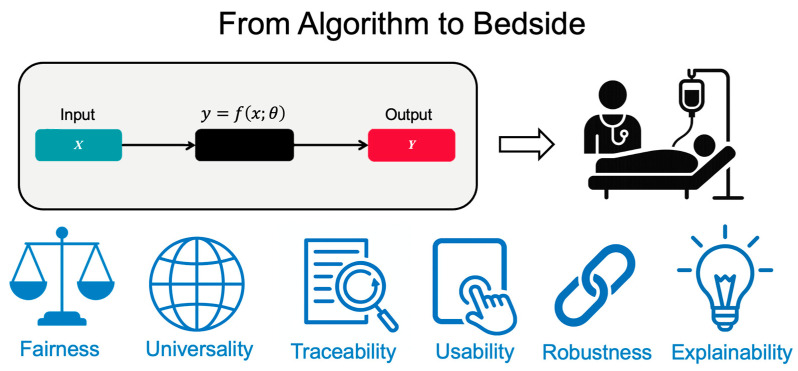
The FUTURE-AI Framework: Essential Requirements for Clinical AI Implementation. The FUTURE-AI framework defines six essential principles for successful bedside implementation: Fairness (equitable performance across populations), Universality (generalization across settings), Traceability (transparent documentation), Usability (workflow integration), Robustness (real-world reliability), and Explainability (interpretable decisions). Each domain has different maximum scores totaling 30 points, providing a quantitative assessment of clinical readiness.

AI helps clinical oncology with both productivity and creativity, and its use will continue to grow. Turning published models into tools that work at the bedside takes more than just accuracy. It also requires trust, fairness, and fit with day-to-day workflows. The FUTURE-AI framework, developed by 117 experts from 50 countries, offers six practical principles to guide this work: fairness, universality, traceability, usability, robustness, and explainability [109] (Figure 5).

### 5.1. Fairness: Ensuring Equitable AI Performance

AI models must perform consistently across all patient populations to avoid perpetuating health disparities [110,111,112,113,114,115,116]. For example, a melanoma detection algorithm trained primarily on light-skinned individuals may show significantly reduced sensitivity for darker-skinned patients, potentially delaying life-saving diagnoses. Similarly, breast cancer detection systems may perform differently based on breast density variations across ethnic groups [117,118]. Achieving fairness requires deliberate strategies throughout development. First, teams must collect representative data across demographic groups, socioeconomic backgrounds, and geographic regions [119]. Federated learning is a powerful solution that enables institutions to collaborate on model training without sharing sensitive patient data. Each hospital trains the model on its local population and shares only encrypted parameter updates. This approach successfully develops algorithms that maintain accuracy across diverse groups [120,121,122]. When disparities are detected, techniques like adversarial debiasing and fairness-aware ML can help correct imbalances while preserving diagnostic accuracy [123].

### 5.2. Universality: Building Models for Diverse Clinical Settings

For AI to transform cancer care globally, models must generalize beyond their development environment [124]. A mammography algorithm developed on equipment from one manufacturer must perform equally well with systems from other vendors. This challenge extends to variations in imaging protocols, patient populations, and even staining techniques in digital pathology [125,126]. Transfer learning provides a critical solution by leveraging knowledge from large, pre-trained models. Rather than requiring millions of oncology-specific images, developers can adapt models originally trained on general photographs to detect lung nodules or classify pathology slides using only thousands of examples [127,128,129]. However, achieving true universality demands more than technical solutions. It also requires standardization efforts across institutions, including harmonized imaging protocols, common data formats, and shared quality metrics that enable models to maintain performance when deployed in new settings [130,131].

### 5.3. Traceability: Creating Transparent AI Systems

The path from training data to clinical decision must be fully documented and auditable [132,133,134,135,136]. Comprehensive traceability serves multiple purposes: enabling rapid identification of problems, facilitating regulatory approval, and building institutional trust. This includes documenting data sources and patient demographics, recording all preprocessing steps and augmentation techniques, tracking model architecture choices and hyperparameter selections, maintaining version control for both code and data, and logging validation results across different test sets. Modern AI platforms increasingly include built-in traceability features that automatically log training runs, data lineage, and performance metrics [137,138]. These systems generate regulatory-ready documentation, streamlining the path from research to clinical deployment. When models fail or show unexpected behavior, this documentation allows for a quick root cause analysis and corrective action.

### 5.4. Usability: Integrating AI into Clinical Workflows

Even the most accurate AI model will fail if oncologists cannot use it effectively [139,140,141]. A lung nodule detection system requiring separate login, manual image upload, and minutes of processing will be adopted only minimally, regardless of its accuracy. Successful integration requires AI to operate seamlessly within existing tools, such as highlighting suspicious regions directly within Picture Archiving and Communication System (PACS) automatically flagging high-risk cases for priority review, and integrating predictions into EHRs. Usability encompasses more than technical integration. AI should reduce, not increase, cognitive load on clinicians. This means presenting information at an appropriate level of detail, providing confidence intervals rather than binary predictions, and allowing easy access to supporting evidence. Development must involve oncologists from the beginning to ensure that interfaces are intuitive and outputs are clinically meaningful. Training materials and hands-on sessions help clinicians understand both the capabilities and limitations of AI.

### 5.5. Robustness: Sustained Performance Under Real-World Conditions

Laboratory performance does not always translate directly to clinical practice [142,143,144,145,146]. Models must maintain accuracy when faced with variations in image quality, missing data, equipment differences, and the full spectrum of real-world complexity. A pathology AI trained on archival-quality slides may fail when confronted with routine clinical specimens that have artifacts, varying stain quality, or tissue folding. Building robust models requires diverse training data that includes edge cases and imperfect examples. Data augmentation techniques, such as artificially introducing noise, rotation, and color variations, help models learn invariance to common perturbations. Continuous monitoring systems detect when models encounter data outside their training distribution or when performance begins to degrade [147,148,149,150,151]. Some systems implement continuous learning, allowing models to adapt to changing practices and populations while preventing “catastrophic forgetting” of previously learned patterns. Furthermore, models must be resilient against deliberate threats, including data poisoning attacks that could corrupt the training data or adversarial attacks designed to cause misclassification at inference time [152]. Safeguarding against these security vulnerabilities is critical for clinical deployment.

### 5.6. Explainability: Making AI Decisions Understandable

The “black box” nature of DL remains a fundamental barrier to clinical trust [153,154,155,156,157]. Oncologists need to understand not only what an AI predicts but also why. This is especially important when recommendations differ from clinical judgment. Explainable AI techniques provide these insights through various methods. For imaging applications, techniques like Grad-CAM generate heatmaps showing which regions influenced a model’s decision, highlighting specific areas of a mammogram that suggest malignancy or pathology features indicative of aggressive disease [158,159,160,161]. For tabular data, SHAP values can rank the importance of different clinical factors and show whether a prognosis was driven primarily by tumor size, patient age, or biomarker status [162,163,164,165]. However, explainability must balance completeness with usability. The most effective systems provide layered explanations, offering simple summaries for routine cases and detailed analyses available when needed while always acknowledging uncertainty and limitations.

### 5.7. Future Directions

Current evidence reveals significant gaps between aspirations and reality. In a systematic review of AI research on radiological imaging for soft-tissue and bone tumors, the mean FUTURE-AI score was only 5.1 out of 30, indicating a substantial gap between current practice and established guidelines [166]. Beyond technical challenges, regulatory frameworks for AI in oncology remain evolving, with significant gaps in liability assignment when AI-assisted decisions lead to adverse outcomes [124]. Key unresolved questions include: Who bears responsibility when AI recommendations cause harm? What constitutes adequate evidence for AI tool approval? These regulatory uncertainties create barriers to implementation and require urgent attention form policymakers, professional societies, and healthcare institutions. Coordinated efforts across multiple stakeholders are required to move forward: developers must incorporate these principles from the outset, institutions must build robust infrastructure for responsible deployment, and regulators must establish frameworks that balance safety with innovation. Most importantly, the oncology community must participate in developing AI systems that address genuine clinical needs, ensuring that artificial intelligence augments—rather than replaces—the physician’s essential role in cancer care. Finally, as model complexity and scale increase, the oncology community should consider the environment cost of computational requirements. Training a single large language model generates emissions equivalent to approximately 300,000 kg of CO_2_, which is roughly five times the lifetime emissions of an average automobile [165,166]. Sustainable AI development requires commitment to algorithmic efficacy, model selection (avoiding unnecessarily large model when smaller ones suffice), and transparent reporting of computational costs. Progress in healthcare must not come at the expense of planetary health.

## 6. Conclusions: Toward Collaborative Intelligence in Oncology

This review traced the evolution of AI in oncology, from traditional ML through DL to transformer-based foundation models, demonstrating its dual impact on enhancing productivity and facilitating creative discovery. While AI can automate diagnostic tasks, streamline workflows, and enable novel insights through computational biomarkers and drug discovery, its successful clinical implementation requires more than technical accuracy. The FUTURE-AI framework emphasizes ensuring fairness, universality, transparency, usability, robustness, and explainability in deployment. Moving forward demands a collaborative approach where AI augments, rather than replaces, clinical judgment, with algorithms handling data-intensive tasks and clinicians providing contextual understanding and personalized care. To achieve this, we need prospective outcome studies, standardization across institutions, and comprehensive education for oncologists. Only through such coordinated efforts can we ensure that AI advances precision medicine while maintaining the patient-centered approach that is fundamental to quality cancer care.

## Abbreviations

The following abbreviations are used in this manuscript:

ADR	Adenoma Detection Rate
AI	Artificial Intelligence
ASCO	American Society of Clinical Oncology
AUROC:	Area Under the Receiver Operating Characteristic Curve
CDK	Cyclin-Dependent Kinase
CNN	Convolutional Neural Network
CT	Computed Tomography
CTNNB1	Catenin Beta 1
CUP	Cancer of Unknown Primary
DL	Deep Learning
EGFR	Epidermal Growth Factor Receptor
EHR	Electronic Health Record
FIGO:	International Federation of Gynecology and Obstetrics
FDA	Food and Drug Administration
Grad-CAM	Gradient-weighted Class Activation Mapping
H&E	Hematoxylin and Eosin
HER2	Human Epidermal Growth Factor Receptor 2
ICI	Immune Checkpoint Inhibitor
IDH	Isocitrate Dehydrogenase
LLM	Large Language Model
MASAI	Mammography Screening with Artificial Intelligence
MDL	Multimodal Deep Learning
ML	Machine Learning
MRI	Magnetic Resonance Imaging
MSI	Microsatellite Instability
mTOR	mammalian target of rapamycin
MUSK	Multimodal transformer with Unified maSKed modeling
NLP	Natural Language Processing
P4	Predictive, Preventive, Personalized, and Participatory
PACS	Picture Archiving and Communication System
PD-L1	Programmed Cell Death Ligand 1
RAG	Retrieval-Augmented Generation
RECIST	Response Evaluation Criteria in Solid Tumors
RNA	Ribonucleic Acid
RNN	Recurrent Neural Network
SCORPIO	Standard Clinical and labOratory featuRes for Prognostication of Immunotherapy Outcomes
SHAP	SHapley Additive exPlanations
SVM	Support Vector Machine
TMB	Tumor Mutational Burden
TNM	Tumor, Node, Metastasis
TOAD	Tumor Origin Assessment via Deep learning
TP53	Tumor Protein p53
UCSF	University of California, San Francisco
WSB	WD repeat and suppressor of cytokine signaling box containing
WSI	Whole-Slide Image
